# Photobiomodulation therapy ameliorates hyperglycemia and insulin resistance by activating cytochrome c oxidase-mediated protein kinase B in muscle

**DOI:** 10.18632/aging.202760

**Published:** 2021-03-26

**Authors:** Longlong Gong, Zhengzhi Zou, Lei Liu, Shuang Guo, Da Xing

**Affiliations:** 1 MOE Key Laboratory of Laser Life Science and Institute of Laser Life Science, South China Normal University, Guangzhou 510631, China; 2 College of Biophotonics, South China Normal University, Guangzhou 510631, China

**Keywords:** photobiomodulation therapy, type 2 diabetes, muscle, cytochrome c oxidase, PTEN/AKT

## Abstract

Ameliorating hyperglycemia and insulin resistance are major therapeutic strategies for type 2 diabetes. Previous studies have indicated that photobiomodulation therapy (PBMT) attenuates metabolic abnormalities in insulin-resistant adipose cells and tissues. However, it remains unclear whether PBMT ameliorates glucose metabolism in skeletal muscle in type 2 diabetes models. Here we showed that PBMT reduced blood glucose and insulin resistance, and reversed metabolic abnormalities in skeletal muscle in two diabetic mouse models. PBMT accelerated adenosine triphosphate (ATP) and reactive oxygen species (ROS) generation by elevating cytochrome *c* oxidase (C*c*O) activity. ROS-induced activation of phosphatase and tensin homolog (PTEN)/ protein kinase B (AKT) signaling after PBMT promoted glucose transporter GLUT4 translocation and glycogen synthase (GS) activation, accelerating glucose uptake and glycogen synthesis in skeletal muscle. C*c*O subunit III deficiency, ROS elimination, and AKT inhibition suppressed the PBMT effects of glucose metabolism in skeletal muscle. This study indicated amelioration of glucose metabolism after PBMT in diabetic mouse models and revealed the metabolic regulatory effects and mechanisms of PBMT on skeletal muscle.

## INTRODUCTION

Photobiomodulation therapy (PBMT) using red to near infrared (630–1000 nm) light has gained attention as a nondestructive and non-damaging physical therapy in ophthalmology, neurology, dermatology, dentology, and regenerative medicine [1]. PBMT is also widely applied to ameliorating and curing diabetic complications, such as diabetic foot [2], diabetic periodontitis [3], and diabetic retinopathy [4]. Recent studies indicate that PBMT improves insulin sensitivity in high-fat diet (HFD)-induced mice [5, 6], reveal that PBMT ameliorates glucose and lipid metabolism disorders in white adipose tissues [5, 6] and 3T3-L1 adipocytes [7] using He-Ne laser (632.8 nm). However, it is unclear whether PBMT reverses metabolic abnormalities of skeletal muscle in type 2 diabetic models.

Skeletal muscle comprises approximately 40% of the total body mass in adults [8] and is responsible for 70–90% of insulin-induced glucose disposal [9, 10]. Glucose is principally stored as glycogen in insulin-stimulated skeletal muscle [9, 10]. Thus, metabolic abnormalities of skeletal muscle severely impair insulin-regulated blood glucose homeostasis. Previous studies showed that insulin resistance of skeletal muscle was the earliest step in the pathogenesis of metabolic syndrome and type 2 diabetes [10, 11]. Normalizing glucose metabolism in skeletal muscle is an important strategy to reduce hyperglycemia and ameliorate whole-body insulin resistance in type 2 diabetes.

PBMT can modulate mitochondrial respiratory chain cytochrome *c* oxidase (C*c*O), increasing mitochondrial membrane potential [12], adenosine triphosphate (ATP) synthesis [13], and reactive oxygen species (ROS) generation [14, 15]. ATP and ROS are important for regulating energy metabolism [16, 17]. An increase in the adenosine monophosphate (AMP)/ATP ratio activates AMP-activated protein kinase (AMPK), which modulates glucose uptake and energy expenditure [17]. Exercise-induced AMPK activity in skeletal muscle enhances glucose uptake and insulin sensitivity [18]. ROS can inactivate protein tyrosine phosphatases (PTPs), such as phosphatase and tensin homolog (PTEN), which negatively regulates the protein kinase B (AKT) signaling pathway [19, 20]. Previous study showed that enhancing transient ROS levels reversed insulin resistance in skeletal muscle [21]. Mice lacking one of the key enzymes involved in the elimination of physiological ROS, glutathione peroxidase 1 (Gpx1), are protected against HFD-induced insulin resistance [22]. Insulin-stimulated AKT activation involves glucose uptake and glycogen synthesis in skeletal muscle [23]. Our previous studies have found photoactivation of AKT in C2C12 cells [24], INS-1 cells [25] and COS-7 cells [26] using He-Ne laser. Thus, PBMT may improve insulin sensitivity of skeletal muscle through AMP/ATP-induced AMPK activation or ROS-induced PTEN/AKT activation in type 2 diabetes.

In the present study, we investigate metabolic regulatory effects of PBMT in high-fat diet (HFD)-fed and diabetic db/db mice and the mechanism behind these effects. We found that C*c*O mediates activation of PTEN/AKT signaling, but not AMPK signaling, in skeletal muscle after PBMT to ameliorate hyperglycemia and insulin resistance in type 2 diabetes. Taken together, this research provides a further demonstration for laser treatment in type 2 diabetes.

## RESULTS

### PBMT reduces blood glucose and insulin resistance in type 2 diabetic mice

**Figure 1:**
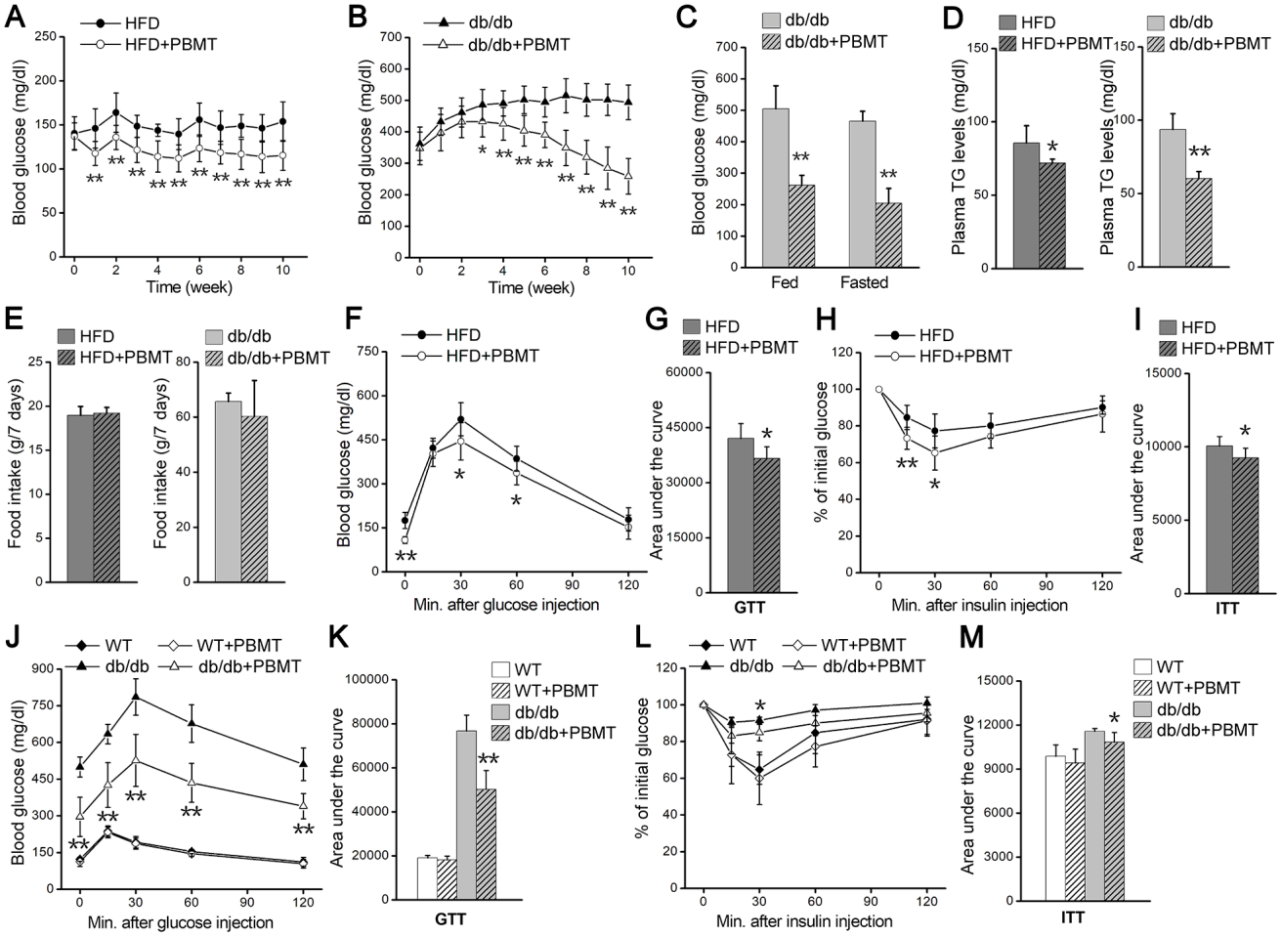
**PBMT reduces blood glucose and insulin resistance in mouse models.** (**A**, **B**) Fasting blood glucose curves of HFD-fed mice (n =7–10) (A) and db/db mice (n = 8–10) (B) with or without PBMT (635 nm, 72.1 mW/cm^2^, 10 min; 8 J/cm^2^) for 10 weeks. Blood glucose was examined 12 hours after the last PBMT. Mean ± SD. *p < 0.05, **p < 0.01 *vs.* the PBMT-untreated mice (Student’s *t*-test). (**C**) Blood glucose in db/db mice treated with or without PBMT for 10 weeks. Blood glucose was examined 12 hours after the last PBMT. Mean ± SD, n = 5. **p < 0.01 *vs.* the db/db mice (Student’s *t*-test). (**D**) Plasma TG levels in HFD-fed mice and db/db mice treated with or without PBMT for 10 weeks. Plasma TG was examined 12 hours after the last PBMT. Mean ± SD, n = 5. *p < 0.05, **p < 0.01 *vs.* the PBMT-untreated mice (Student’s *t*-test). (**E**) Food intake for 7 days in PBMT-untreated or -treated HFD-fed mice and db/db mice for 10 weeks. Mean ± SD, n = 5. (**F**, **G**) GTTs (**F**) and area under the curve (above baseline glucose in GTTs) (**G**) in HFD-fed mice treated with or without PBMT for 10 weeks. The experiences were performed 12 hours after the last PBMT. Mean ± SD, n = 7. *p < 0.05, **p < 0.01 *vs.* the HFD mice (Student’s *t*-test). (**H**, **I**) ITTs (**H**) and area under the curve (above baseline glucose in ITTs) (**I**) in HFD-fed mice treated with or without PBMT for 10 weeks. The experiences were performed 12 hours after the last PBMT. Mean ± SD, n = 7. *p < 0.05, **p < 0.01 *vs.* the HFD mice (Student’s *t*-test). (**J**, **K**) GTTs (**J**) and area under the curve (**K**) in wild type (WT) and db/db mice treated with or without PBMT for 10 weeks. The experiences were performed 12 hours after the last PBMT. Mean ± SD, n = 5. **p < 0.01 *vs.* the db/db mice (Student’s *t*-test). (**L**, **M**) ITTs (**L**) and area under the curve (**M**) in WT and db/db mice with or without PBMT for 10 weeks. The experiences were performed 12 hours after the last PBMT. Mean ± SD, n = 5. *p < 0.05 *vs.* the db/db mice (Student’s *t*-test).

To explore biological effects of PBMT in type 2 diabetes, we examined the changes in blood glucose in HFD-fed and db/db diabetic mice after PBMT. Blood glucose and body weight in mice fed a high-fat diet for 5 weeks were significantly increased compared with the normal-chow diet (NCD)-fed group (Supplementary Figure 1B, Supplementary Figure 1C). After PBMT, the fasting blood glucose in HFD-fed mice was decreased compared with the untreated group (Figure 1A). We next examined the metabolic regulatory effects of PBMT in diabetic db/db mice. Blood glucose and body weight in 6-week-old db/db mice were significantly higher than wild-type mice (Supplementary Figure 1D, Supplementary Figure 1E). Consistent with HFD-fed diabetic mice, fasting and fed blood glucose levels were decreased in db/db mice that received PBMT for 10 weeks (Figure 1B, 1C). However, there was no change in blood glucose in wild-type mice treated with or without PBMT (Supplementary Figure 1F). Meanwhile, plasma triglyceride (TG) levels were reduced in both HFD-fed and db/db mice after PBMT (Figure 1D).

To explore the reason for the decreased blood glucose after PBMT, mouse food and water intake were monitored. There was no difference in food intake between the control and laser-treated groups (Figure 1E). Although water intake was unchanged in HFD-fed mice (Supplementary Figure 1G), both water intake and urinary output were decreased in db/db mice after PBMT (Supplementary Figure 1G, Supplementary Figure 1H). Since skeletal muscle is responsible for most insulin-induced glucose disposal [9, 10], the metabolic improvements might be because PBMT ameliorated metabolic abnormalities in skeletal muscle.

Next, whole-body insulin resistance was assessed using glucose tolerance test (GTT) and insulin tolerance test (ITT) in both mouse models in response to PBMT. In HFD-fed mice, laser-treated mice exhibited better glucose tolerance than the untreated group (Figure 1F, 1G). Insulin sensitivity was also markedly enhanced in the PBMT HFD-fed mice (Figure 1H, 1I). Similarly, glucose tolerance and insulin sensitivity were improved in db/db mice after PBMT, whereas there was no difference in wild-type (WT) mice (Figure 1J–1M). These findings suggest that PBMT could ameliorate whole-body insulin resistance in both HFD-fed and db/db mice.

### PBMT ameliorates metabolic disorders in skeletal muscle

**Figure 2:**
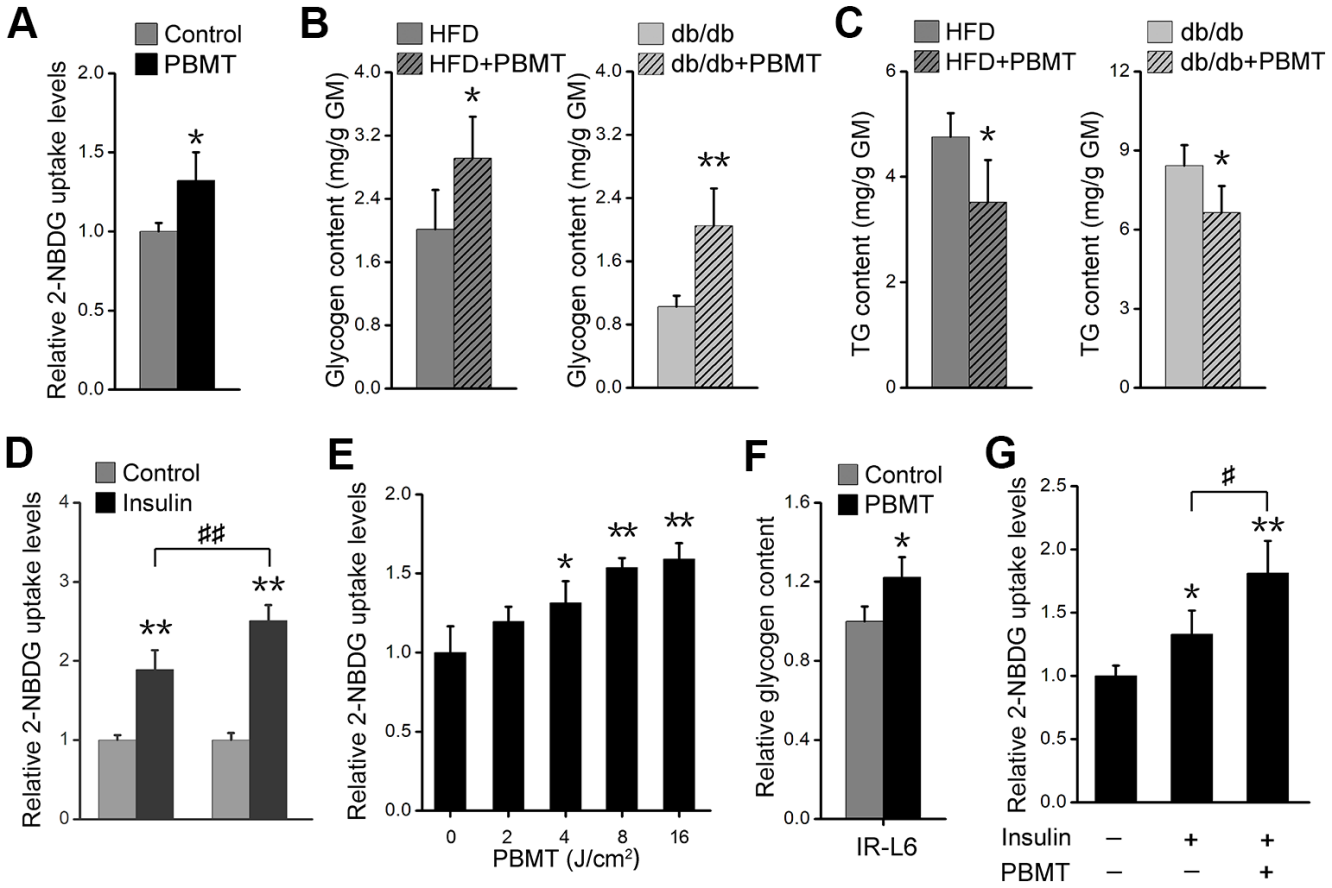
**PBMT ameliorates metabolic disorders of skeletal muscle in mouse models.** (**A**) 2-NBDG uptake of GMs 30 min after PBMT. The fresh muscles were isolated from HFD-fed mice for 6 weeks. Mean ± SD, n = 4. *p < 0.05 *vs.* the control group (Student’s *t*-test). (**B**, **C**) Glycogen (**B**) and TG (**C**) contents in GMs isolated from HFD-fed mice and db/db mice with or without PBMT for 10 weeks. The experiences were performed 12 hours after the last PBMT. Mean ± SD, n = 4. *p < 0.05, **p < 0.01 *vs.* the PBMT-untreated mice (Student’s *t*-test). (**D**) 2-NBDG uptake in insulin-stimulated GM from HFD-fed mice with or without PBMT for 10 weeks. The experiences were performed 12 hours after the last PBMT. Mean ± SD, n = 4. **p < 0.01 *vs.* the control groups; ##*p* < 0.01 *vs.* the indicated group (Student’s *t*-test). (**E**) 2-NBDG uptake in IR-L6 myotubes 30 min after different doses of laser irradiation. Mean ± SD, n = 4. *p < 0.05, **p < 0.01 *vs.* the PBMT-untreated group (Student’s *t*-test). (**F**) Glycogen content in IR-L6 myotubes treated with 8 J/cm^2^ PBMT every 12 h for 1 day. Six hours after the last PBMT, glycogen was measured. Mean ± SD, n = 4. *p < 0.05 *vs.* the control group (Student’s *t*-test). (**G**) 2-NBDG uptake in IR-L6 myotubes 30 min after 10 nM insulin and/or 8 J/cm^2^ PBMT. Mean ± SD, n = 4. *p < 0.05, **p < 0.01 *vs.* the control group; #*p* < 0.05 *vs.* the indicated group (Student’s *t*-test).

Muscle is one of the principal subcutaneous tissues [10, 27]. During PBMT, a 635-nm laser penetrates the skin and enters muscle tissue; therefore, we investigated whether the light regulates glucose metabolism in skeletal muscle. PBMT markedly promoted nonradioactive fluorescent glucose 2-(N-(7-nitrobenz-2-oxa-1,3-diazol-4-yl)amino)-2-deoxyglucose (2-NBDG) uptake in gastrocnemius muscle (GM) (Figure 2A). Muscle glycogen content was also increased in GM isolated from HFD-fed and db/db mice after PBMT (Figure 2B). However, ectopic fat deposition was significantly attenuated in GM isolated from the laser-treated mouse models (Figure 2C). The improved metabolic abnormalities in skeletal muscle indicate that PBMT may strengthen muscle function in terms of blood glucose disposal in response to insulin stimulation. The 2-NBDG uptake abilities of insulin-stimulated GMs were significantly enhanced from the laser-treated mice compared with the untreated groups (Figure 2D, Supplementary Figure 2A). Thus, PBMT improved insulin sensitivity in insulin-resistant skeletal muscle.

Further, we built an insulin-resistant L6 (IR-L6) myotube model (Supplementary Figure 2B–2D). The results showed that 2-NBDG uptake was increased in IR-L6 myotubes after PBMT (Figure 2E). Increased glucose consumption in Dulbecco’s modified Eagle’s medium (DMEM) was also detected in IR-L6 myotubes after PBMT (Supplementary Figure 2E). PBMT promoted intracellular glycogen synthesis in IR-L6 myotubes (Figure 2F). These results were consistent with the skeletal muscle from HFD and db/db mice. Meanwhile, co-treatment with both PBMT and insulin increased intracellular 2-NBDG levels in IR-L6 myotubes compared with the insulin only-treated group (Figure 2G), which suggested that PBMT improved insulin sensitivity in IR-L6 myotubes. Together, these data indicate that PBMT could accelerate glucose absorption and glycogen synthesis in insulin-resistant skeletal muscles *via* a mechanism involving a laser-induced reduction in blood glucose in HFD-fed and db/db mice.

### C*c*O mediates PBMT-enhanced ATP and ROS production

**Figure 3:**
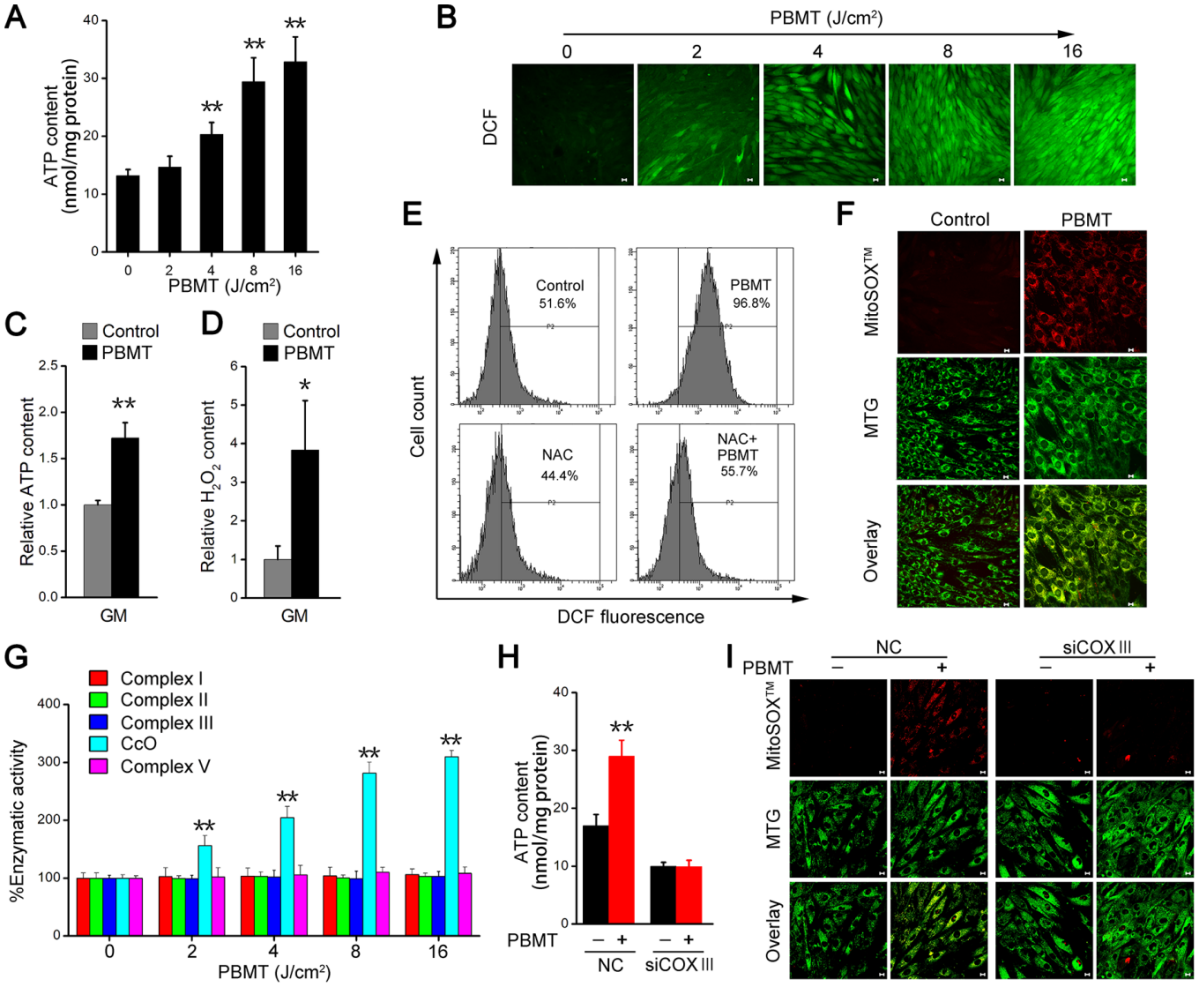
**PBMT facilitates ATP and ROS generation by increasing mitochondrial C*c*O activity.** (**A**) Intracellular ATP content in IR-L6 myotubes 15 min after the indicated doses of PBMT. Mean ± SD, n = 4. **p < 0.01 *vs.* the PBMT-untreated group (Student’s *t*-test). (**B**) Monitoring of ROS generation stimulated by the indicated doses of PBMT in IR-L6 myotubes. ROS (green) was stained with H_2_DCFDA. Scale bar, 10 μm. (**C**, **D**) Intracellular ATP (**C**) and H_2_O_2_ (**D**) content in GMs 15 min after 8 J/cm^2^ PBMT. Mean ± SD, n = 4. **p < 0.01 *vs.* the control group (Student’s *t*-test). (**E**) Flow cytometry (FACS) analysis of ROS generation in IR-L6 myotubes 15 min after the indicated treatments. Cells were pre-cultured with N-acetylcysteine (NAC; 250 μM) 1 h before PBMT. Mean ± SD, n = 3. *p < 0.05, **p < 0.01 *vs.* the control group; ##*p* < 0.01 *vs.* the indicated group (Student’s *t*-test). (**F**) Representative fluorescence images of O_2_^•–^ (red) and mitochondria (green) in IR-L6 myotubes treated with or without 8 J/cm^2^ PBMT. O_2_^•–^ was stained with MitoSOX^TM^; mitochondria were stained with MitoTracker Green (MTG). Scale bar, 10 μm. (**G**) Enzymatic activity of Complex I, II, III, C*c*O, and V in IR-L6 myotubes after the indicated doses of PBMT. Mean ± SD, n = 4. **p < 0.01 *vs.* the PBMT-untreated group (Student’s *t*-test). (**H**, **I**) Intracellular ATP content (**H**) and representative fluorescence images of O_2_^•–^ (red) and mitochondria (green) (**I**) in IR-L6 myotubes transfected with negative-control siRNA (NC) or COXIII siRNA (siCOXIII) 15 min after 8 J/cm^2^ PBMT. Mean ± SD, n = 4. **p < 0.01 *vs.* the PBMT-untreated groups (Student’s *t*-test). Scale bar, 10 μm.

We next examined the mechanisms mediating the metabolic improvements induced by PBMT in insulin-resistant skeletal muscles. PBMT promoted ATP and ROS generation in IR-L6 myotubes (Figure 3A, 3B), as well as in GM (Figure 3C, 3D). N-acetylcysteine (NAC), a ROS scavenger, dramatically decreased the intracellular ROS content in IR-L6 myotubes after PBMT (Figure 3E). Superoxide anion O2^•–^ is a precursor of most other ROS [28]; high levels of O2^•–^ were generated and completely co-localized with mitochondria in IR-L6 myotubes after PBMT (Figure 3F). These results indicate that PBMT increased intracellular ATP and ROS levels in insulin-resistant skeletal muscles.

The mitochondrial respiratory chain is an important source of ATP and ROS. We next detected enzymatic activity of mitochondrial respiratory chain complexes, including Complex I, II, III, C*c*O, and V. The results showed that the enzymatic activity of C*c*O was enhanced in a laser dose-dependent manner in IR-L6 myotubes and the enzymatic activity of Complex I, II, III, V was no change in between PBMT-treated and untreated groups (Figure 3G). In C*c*O subunit III (COXIII)-deficient IR-L6 myotubes (Supplementary Figure 3A), the increased ATP and O2^•–^ generation after PBMT was blocked (Figure 3H, 3I). Similarly, sodium azide (NaN_3_), a C*c*O inhibitor, also inhibited PBMT-generated ATP and O2^•–^ in IR-L6 myotubes (Supplementary Figure 3B, Supplementary Figure 3C). These results demonstrate that PBMT facilitated mitochondrial ATP and O2^•–^ generation by increasing the enzymatic activity of C*c*O in insulin-resistant skeletal muscle.

### PBMT-induced ROS activates the PTEN/AKT pathway

**Figure 4:**
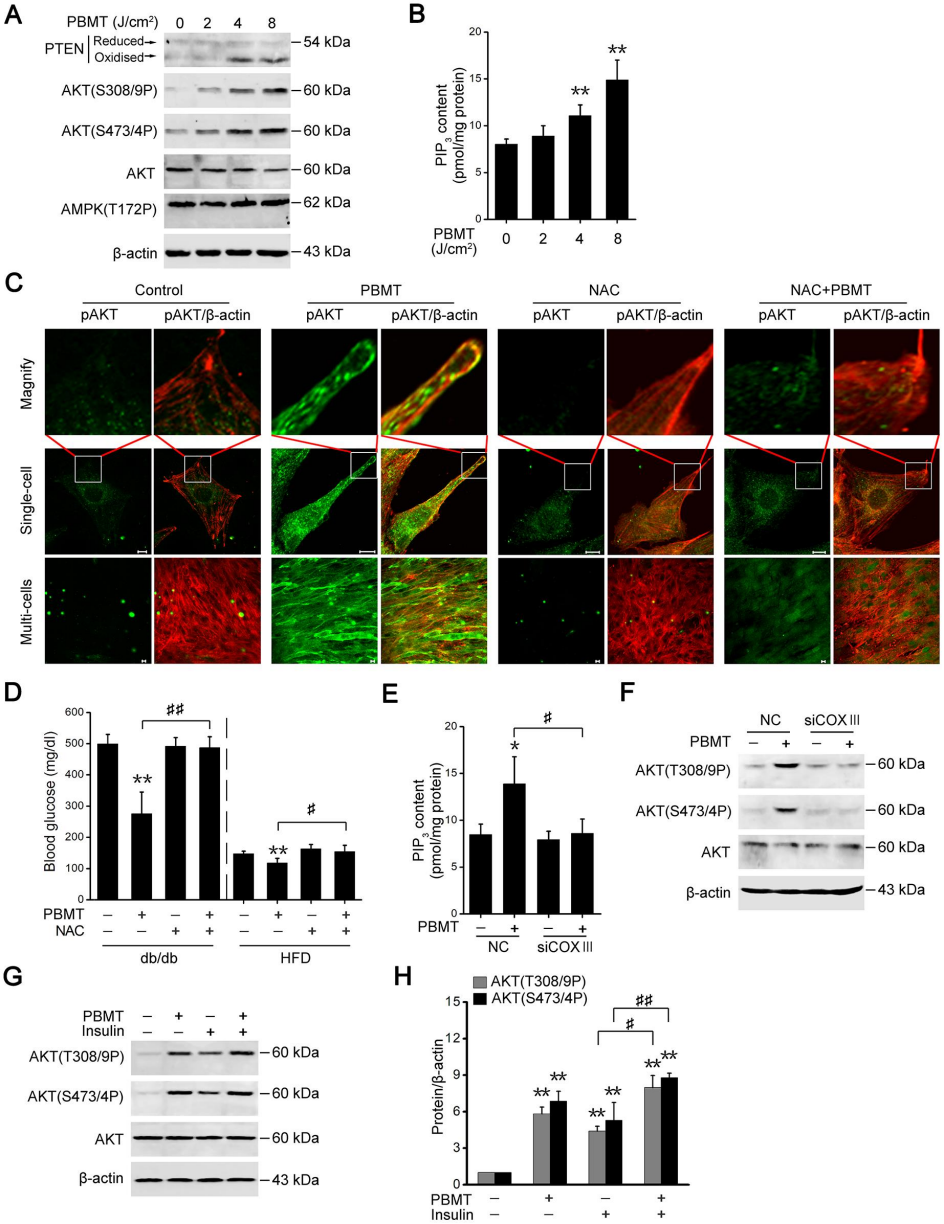
**PBMT-increased ROS but not ATP improves insulin sensitivity by activating the PTEN/AKT signaling.** (**A**) Immunoblot analysis of PTEN oxidation, AKT phosphorylation and AMPK phosphorylation in IR-L6 myotubes 30 min after different doses of laser irradiation. (**B**) Intracellular PIP_3_ levels in IR-L6 myotubes 30 min after different doses of laser irradiation. Mean ± SD, n = 4. **p < 0.01 *vs.* the PBMT-untreated group (Student’s *t*-test). (**C**) Representative immunofluorescence images of AKT(S473/4P) (green) and β-actin (red) in IR-L6 myotubes under the indicated treatments. Scale bar, 10 μm. (**D**) Fasting blood glucose in db/db mice and HFD-fed mice with the indicated treatments for three weeks and one week, respectively. Mice were injected intraperitoneally once daily with NAC (100 mg/kg) 30 min before each PBMT. Blood glucose was examined 12 hours after the last PBMT. Mean ± SD, n = 5. **p < 0.01 *vs.* the control groups; #*p* < 0.05, ##*p* < 0.01 *vs.* the indicated groups (Student’s *t*-test). (**E**, **F**) PIP_3_ content (n = 4) (**E**) and AKT phosphorylation (**F**) in IR-L6 myotubes transfected with NC or COXIII siRNA under the indicated treatments. The experiment was performed 30 min after PBMT. Mean ± SD. *p < 0.05 *vs.* the control groups; #*p* < 0.05 *vs.* the indicated group (Student’s *t*-test). (**G**, **H**) Immunoblot analysis (**G**) and gray analysis (**H**) of AKT phosphorylation in IR-L6 myotubes 30 min after PBMT or/and insulin treatments. Mean ± SD, n = 3. **p < 0.01 *vs.* the untreated groups; #*p* < 0.05, ##*p* < 0.01 *vs.* the indicated groups (Student’s *t*-test).

Activation of AMPK signaling depends on an increase in the intracellular AMP/ATP ratio [29]; however, PBMT increased ATP production, suggesting that PBMT did not activated AMPK signaling in insulin-resistant skeletal muscles. As shown in Figure 4A, there was no change in phosphorylation of AMPK at Thr172 in IR-L6 myotubes after PBMT. But PTEN oxidation and AKT phosphorylation were increased in IR-L6 myotubes after PBMT (Figure 4A). Meanwhile, increased intracellular phosphatidylinositol (3,4,5)-trisphosphate (PIP_3_) contents were detected in IR-L6 myotubes after PBMT (Figure 4B). Immunofluorescence staining showed that PBMT promoted AKT activation and translocation to the cytomembrane, but NAC inhibited these effects in IR-L6 myotubes after PBMT (Figure 4C).

Consistent with the *in vitro* findings, the effects of laser treatment on reducing blood glucose levels were suppressed in diabetic model mice preinjected with NAC (Figure 4D). These data suggest that PBMT could activate AKT by stimulating ROS generation in insulin-resistant skeletal muscle. In COXIII-knockdown IR-L6 myotubes, the ability of PBMT to increase PIP_3_ contents and AKT phosphorylation was inhibited (Figure 4E, 4F). These data demonstrate that C*c*O mediated the photoactivation of PTEN/AKT signaling in insulin-resistant skeletal muscle.

AKT phosphorylation was also increased in the PBMT and insulin co-treated IR-L6 myotubes compared with insulin only-treated myotubes (Figure 4G, 4H). These data indicate that PBMT improved insulin sensitivity by enhancing activation of PTEN/AKT signaling in insulin-resistant skeletal muscle.

### PBMT-activated AKT signaling promotes GLUT4 translocation

**Figure 5:**
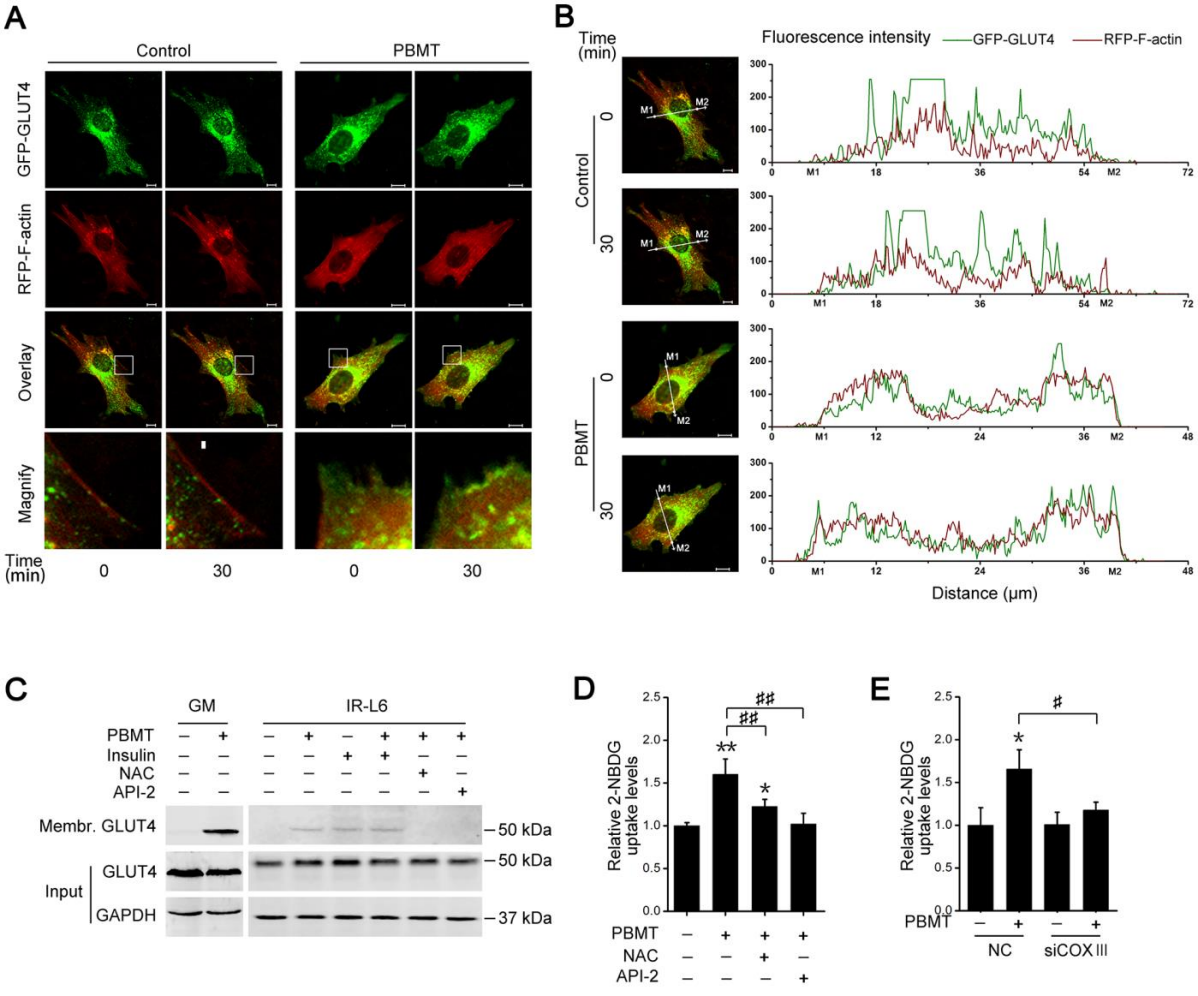
**Activation of AKT signaling promotes glucose transporter GLUT4 translocation after PBMT.** (**A**, **B**) Representative images (**A**) of GFP-GLUT4 (green) subcellular localization (red, RFP-F-actin) were obtained in IR-L6 myotubes treated with or without 8 J/cm^2^. The red areas of the RFP-F-actin images show the cell contour. Fluorescence intensity (**B**) of GFP-GLUT4 and RFP-F-actin along the white lines in fluorescence images of IR-L6 myotubes. M1 and M2 are cell boundaries. Scale bar, 10 μm. (**C**) Immunoblot analysis of GLUT4 in the cell membrane fraction of fresh GM and IR-L6 myotubes 30 min after 8 J/cm^2^ PBMT or 10 nM insulin treatment. Cells were pre-cultured with NAC (250 μM) or API-2 (2 μM) 1 hour before PBMT. (**D**) 2-NBDG uptake in IR-L6 myotubes 30 min after PBMT in the presence of either NAC or API-2. Mean ± SD, n = 3. *p < 0.05, **p < 0.01 *vs.* the PBMT-untreated group; ##*p* < 0.01 *vs.* the indicated group (Student’s *t*-test). (**E**) 2-NBDG uptake in IR-L6 myotubes transfected with NC or COXIII siRNA 30 min after the indicated treatments. Mean ± SD, n = 3. *p < 0.05 *vs.* the PBMT-untreated group; #*p* < 0.05 *vs.* the indicated group (Student’s *t*-test).

The data presented above show that PBMT promotes glucose uptake and activates AKT signaling in insulin-resistant muscle. The glucose transporter GLUT4 is regulated by activated AKT signaling in skeletal muscle [30, 31]. To assess whether PBMT induced the translocation of GLUT4 to the cell membrane, IR-L6 myotubes were transfected with a GFP-GLUT4 expression plasmid. PBMT promoted the accumulation of GFP-GLUT4 at the cell surface in IR-L6 myotubes (Figure 5A, 5B). There was significantly more GLUT4 protein in the cell membrane fraction of laser-irradiated GM and IR-L6 myotubes (Figure 5C). Treatment with either the ROS scavenger NAC or the AKT inhibitor API-2 suppressed PBMT-increased GLUT4 translocation in IR-L6 myotubes (Figure 5C). Meanwhile, laser-increased glucose uptake was inhibited in IR-L6 myotubes by pretreatment with NAC or API-2 (Figure 5D). Similarly, COXIII deficiency also blocked the laser effect in IR-L6 myotubes (Figure 5E). Thus, PBMT promoted GLUT4 translocation and glucose uptake in insulin-resistant skeletal muscles by inducing the photoactivation of C*c*O-mediated AKT signaling.

### PBMT-activated AKT signaling restores glycogen synthase activation

**Figure 6:**
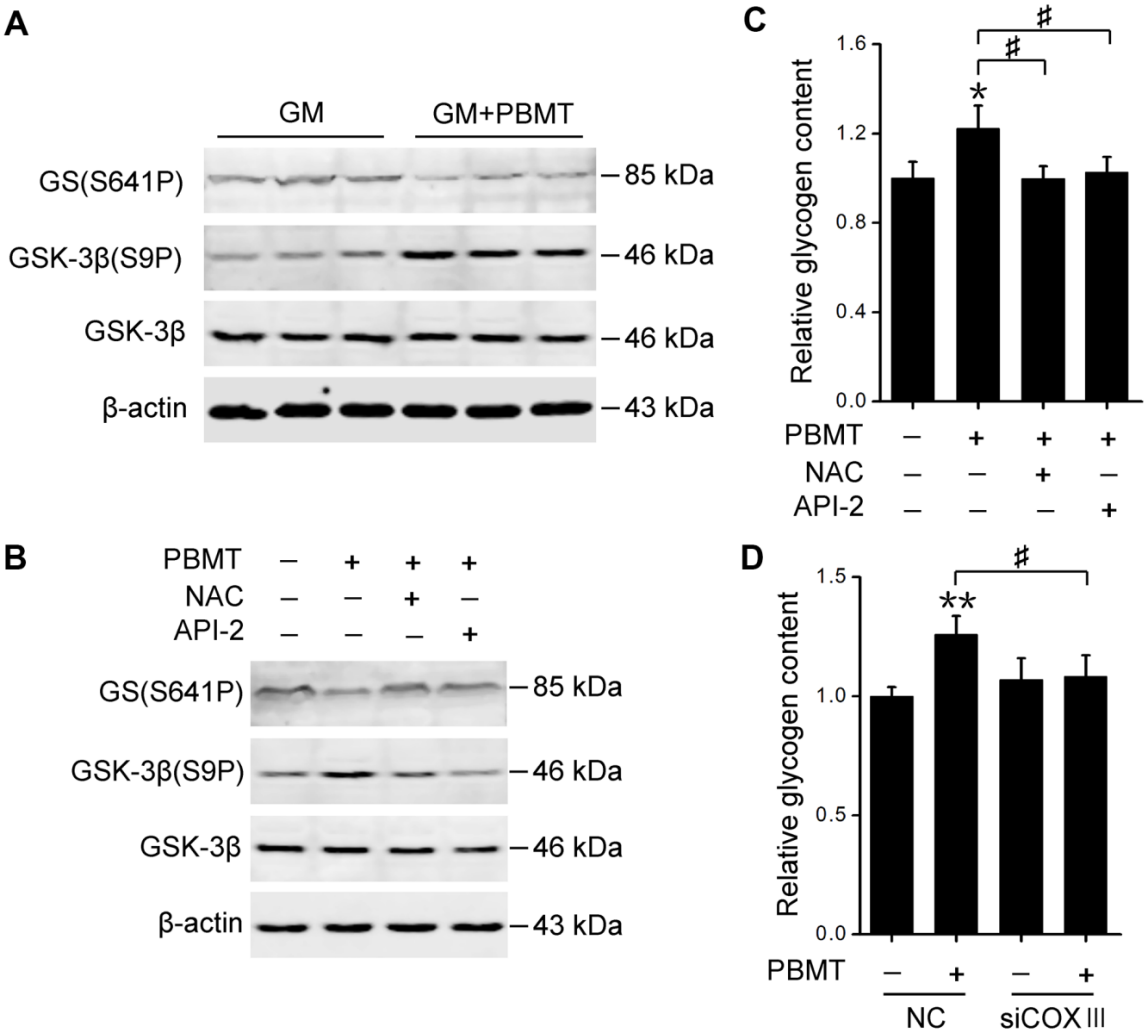
**Activation of AKT signaling restores GS activity in skeletal muscle after PBMT.** (**A**, **B**) Immunoblot analysis of GSK-3β(S9P) and GS(S641P) in GM (**A**) and IR-L6 myotubes (**B**) 30 min after the indicated treatments. (**C**) Glycogen content was measured in IR-L6 myotubes 30 min after 8 J/cm^2^ PBMT in the presence of either NAC or API-2 every 12 h for 1 day. Mean ± SD, n = 4. *p < 0.05 *vs.* the PBMT-untreated group; #*p* < 0.05 *vs.* the indicated groups (Student’s *t*-test). (**D**) Glycogen content in IR-L6 myotubes transfected with NC or COXIII siRNA. The myotubes treated with 8 J/cm^2^ PBMT every 12 h for 1 day. Six hours after the last PBMT, glycogen was measured. Mean ± SD, n = 4. **p < 0.01 *vs.* the PBMT-untreated group; #*p* < 0.05 *vs.* the indicated group (Student’s *t*-test).

Finally, we explored the mechanism behind PBMT-increased glycogen synthesis in insulin-resistant skeletal muscles. Glycogen synthase (GS) activation was examined, which is phosphorylated and inactivated by glycogen synthase kinase-3β (GSK-3β) [32]. PBMT increased GSK-3β phosphorylation and reduced GS phosphorylation in GM and IR-L6 myotubes, suggesting that laser treatment restored GS activity by inhibiting GSK-3β (Figure 6A, 6B). Treatment with either a ROS scavenger or AKT inhibitor suppressed laser-stimulated GS activation and glycogen accumulation in IR-L6 myotubes (Figure 6B, 6C). COXIII deficiency also blocked laser-increased glycogen synthesis in IR-L6 myotubes (Figure 6D). These data indicate that C*c*O photoactivation-mediated AKT signaling promotes GS activation and glycogen synthesis, ameliorating glucose metabolism in insulin-resistant skeletal muscles.

## DISCUSSION

Many biophysical (i.e., electrical, magnetic, and ultrasound) methods are being explored for their ability to prevent and ameliorate diseases. The ability of PBMT to activate endogenous protein signaling has been used to direct the treatment of many diseases in ophthalmology, neurology, dermatology, dentology, and regenerative medicine [1]. The present study found that PBMT reduced blood glucose and insulin resistance by increasing glucose uptake and glycogen synthesis in the skeletal muscle of HFD-fed and db/db mice. In addition, PBMT accelerated mitochondrial ATP and ROS generation by increasing mitochondrial respiratory chain C*c*O activity. Increased ATP generation might elevate lipid consumption and attenuated fat deposition in skeletal muscle, whereas the transient ROS production induced by PBMT promoted activation of the PTEN/AKT/GLUT4 and PTEN/AKT/GSK-3β/GS pathways, increasing glucose absorption and glycogen accumulation in skeletal muscle. PBMT also ameliorates glucose and lipid metabolism disorders in white adipose tissues and 3T3-L1 adipocytes [5–7]. Although in the present study we cannot exclude effects of PBMT on adipose tissues, improved glucose metabolism in skeletal muscle helped reduce hyperglycemia and whole-body insulin resistance in type 2 diabetes.

Several studies have investigated the underlying molecular mechanisms on PBMT-induced biological effects. The molecular mechanisms have mainly focused on mitochondrial respiratory chain: laser irradiation promotes ROS generation by modulating C*c*O [14, 15, 33]. Prof. Wharton and Tzagaloff have revealed that the copper absorption band is between 700 and 1000 nm, the heme gamma absorption band is around 430 nm and the heme alpha band is around 603 nm cytochrome oxidase from beef heart early in 1964 [34]. Although light absorption of purified C*c*O was not examined at 635 nm, activation of C*c*O was observed in skeletal muscle and cells in the present study. Activation of C*c*O accelerates the transfer of electrons from cytochrome c to molecular oxygen, increasing mitochondrial membrane potential and ATP and ROS generation [1]. No significant changes were observed in Complexes I, II, III and V activity after PBMT. Our previous work also indicated the similar results in SH-SY5Y-APPswe cells after He-Ne laser treatment [35]. Inhibition or knockdown of C*c*O blocked PBMT-increased ATP and ROS generation, and PBMT-induced metabolic regulation in insulin-resistant muscle. C*c*O and Complexes I, II, III and V together constitute the complete oxidative phosphorylation system. Thus we cannot exclude the effects on function of Complexes I, II, III and V after inhibition and knockdown of C*c*O.

ROS, important physiological regulators of insulin signaling, are generated at the plasma membrane and endomembranes by NADPH oxidase in skeletal muscle after insulin treatment [16, 36]. Insulin-generated ROS inactivate PTEN by oxidizing cysteine residues in its active site, suppress PTEN-dephosphorylated PIP_3_ to enhance the PI3-K/AKT signaling pathway [20]. Although generation of O2^•–^ were first observed in mitochondria after PBMT, PBMT-generated ROS diffused in the whole IL-L6 myotubes. Intracellular ROS also oxidized and inactivated PTEN, activating AKT signaling in insulin-resistant skeletal muscle after PBMT. Similarly, Gpx1 deficiency in cells and mice inhibits the elimination of intracellular ROS and enhances insulin sensitivity [22]. Aerobic exercise promotes mitochondrial ROS generation and increases insulin sensitivity of skeletal muscle in type 2 diabetes [37, 38], which suggests that ROS involve importantly in insulin signaling regulation in skeletal muscle.

In conclusion, we revealed the effects and molecular mechanisms of PBMT on modulating glucose metabolism. PBMT reduced hyperglycemia and insulin resistance by ameliorating metabolic abnormalities of skeletal muscle in diabetic mouse models. Photoactivation of C*c*O-mediated PTEN/AKT signaling promoted GLUT4 translocation and GS activation, increasing glucose absorption and glycogen synthesis in insulin-resistant skeletal muscle. Thus, PBMT, as a non-oral and noninvasive treatment, may have great value to control type 2 diabetes.

## MATERIALS AND METHODS

### Mice

C57BL/6J mice were purchased from Beijing Huafukang Bioscience (Beijing, China). Male C57BLKS/J-lepr^db^/lepr^db^ diabetic (db/db) mice were purchased from the Jackson Laboratory (Maine, USA). Mice were maintained in a pathogen-free animal facility with a normal chow diet (5% fat; Beijing Huafukang Bioscience, Beijing, China) and 12-hour light-dark cycles. For HFD experiments, 5-week-old C57BL/6J mice (n = 20) were fed a high-fat diet (60% fat, 20% carbohydrate, 20% protein; D12492, Beijing Huafukang Bioscience, Beijing, China) for the indicated time. And 5-week-old C57BL/6J mice (n = 12) were fed a normal chow diet. All studies were performed in accordance with the guidelines of The Care and Use of Laboratory Animals (Institute of Laboratory Animal Resources, Commission on Life Sciences, National Research Council) and approved by The Institutional Animal Care and Use Committee of The South China Normal University, Guangzhou, China.

### Cell lines and tissues culture

L6 myoblasts were obtained from American Type Culture Collection (ATCC, Manassas, USA), and cultured in high-glucose DMEM (Gibco, Grand Island, USA) containing 10% fetal bovine serum (FBS; Gibco, Grand Island, USA), penicillin (100 U/mL), and streptomycin (100 μg/mL) in a humidified incubator containing 5% CO_2_ at 37° C.

For L6 myoblast differentiation, 80% confluent cells were transferred to DMEM with 2% horse serum (Gibco, Grand Island, USA) for 4–6 days. L6 myotubes were used 4–6 days after the initiation of differentiation (Supplementary Figure 2A). For insulin resistance, differentiated IR-L6 myotubes were induced in DMEM containing 2% horse serum plus 100 nM insulin for 48 h [39]. The insulin-stimulated AKT phosphorylation (Supplementary Figure 2B) and 2-NBDG uptake (Supplementary Figure 2C) in IR-L6 myotubes were significantly reduced as compared with in normal L6 myotubes.

GM was isolated from mice, washed with PBS, and incubated for 30 min in Krebs-Henseleit buffer (KHB) containing 0.5% BSA, 2 mM sodium pyruvate, and 6 mM mannitol in a humidified incubator (5% CO_2_, 37° C).

### PBMT treatment

Mouse experiments were conducted as described previously [40, 41]. The body hair of each mouse was removed using 0.8 g/l NaS once every 4 days. HFD-fed mice (n = 10) for five weeks and 6-week-old db/db mice (n = 10) received PBMT. PBMT was administered from 6:00 p.m. to 8:00 p.m. once daily for 10 weeks and room temperature was maintained at 21° C. The mice were fixed in a flat, barrel-shaped transparent device and irradiated in the dark with Laser Diode (wavelength: 635 nm, Output power: 5 W, FCM638M5KLC1P6; Fiblaser, Shanghai, China) with a power density of 72.1 mW/cm^2^ for 10 min to obtain an energy density of 43.3 J/cm^2^. Specifications for irradiation and treatment parameters were listed in Supplementary Tables 1–3. Subcutaneous muscle tissues received an energy density of 8 J/cm^2^ (Supplementary Figure 1A). There was no significant change in the temperature of the skin surface throughout the laser irradiation. The control mice were maintained in a specially designed holder for the same amount of time used as the irradiated groups, but the laser source was not activated (sham irradiation). For NAC treatment, mice were injected intraperitoneally (i.p.) once daily with NAC (100 mg/kg, Sigma, St. Louis, MO, USA) 30 min before the laser treatment until the termination of the experiments.

For tissues and cells, experiments of the laser irradiation were conducted as described previously [42]. GMs were isolated from mice euthanized by cervical dislocation. A549 cells, ρ^0^A549 cells, IR-L6 myotubes and GM were illuminated with the Laser Diode with power densities of 6.67, 13.3, 26.7, and 53.3 mW/cm^2^ for 5 min in the dark, with energy densities of 2, 4, 8, and 16 J/cm^2^, respectively. The control groups were maintained in a specially designed holder for the same amount of time used as the irradiated groups, but the laser source was not activated (sham irradiation).

### Metabolic studies and bioassays

Fed and fasting after a 12-hour (overnight) blood glucose was measured using an automatic glucose monitor (ACCU-Check Active, Roche Diagnostics GmbH, Germany). Food and water intake within 7 days were measured in mice with PBMT for 10 weeks. Urinary output within 3 days was detected in mice with PBMT for 10 weeks. The mouse was singly caged.

ATP, TG, PIP_3_ and glycogen in GMs isolated from PBMT-irradiated mice, L6 myotubes and A549 cells after different treatments were measured by using an ATP Assay Kit (Beyotime, Shanghai, China), TG assay kit (GPO-POD; Applygen Technologies Inc., Shanghai, China), PIP_3_ ELISA kit (Enzyme-linked Biotechnology Co., Shanghai, China) and glycogen ELISA kit (Enzyme-linked Biotechnology Co., Shanghai, China), respectively, according to the manufacturers’ instructions. Pretreatment with 1 mM NaN_3_ 1 h before PBMT was performed in IR-L6 myotubes. In addition, plasma TG levels in HFD-fed mice and db/db mice after a 12-hour (overnight) fasting were measured.

### Glucose- and insulin- tolerance tests

Glucose- and insulin- tolerance tests were performed as follows [43, 44]. For GTTs, mice were injected intraperitoneally with 20% glucose (2 g/kg) after 16 h fasting, then blood glucose was monitored at 0, 15, 30, 60 and 120 min. For ITTs, insulin (0.6 U/kg) was injected intraperitoneally after 4 h fasting, then blood glucose was measured at 0, 15, 30, 60 and 120 min.

### Glucose uptake assays

Glucose uptake was measured using nonradioactive fluorescent glucose 2-NBDG (Sigma, St. Louis, MO, USA), as described previously [45]. For skeletal muscle experiments, fresh GM from mice after insulin or/and PBMT was incubated with 2-NBDG (50 μM) for 30 min. The specimens were washed and lysed, and 2-NBDG levels in GM were quantified using a microplate fluorimeter (Infinite M200; Tecan, Hillsborough, NC, USA). For cell experiments, IR-L6 myotubes were incubated in KHB containing 50 μM 2-NBDG with or without 8 J/cm^2^ PBMT at 37° C for 30 min. Cells were lysed, then 2-NBDG (Ex/Em, 465/540 nm) was quantified with a microplate fluorimeter. For glucose consumption in DMEM medium, IR-L6 myotubes were serum-starved for 12 hours and irradiated by PBMT. The cells were cultured in fresh free-serum medium in a humidified incubator containing 5% CO_2_ at 37° C. After 12 hours, glucose consumption in the DMEM medium was measured using Glucose Oxidase Method (Applygen Technologies Inc., Shanghai, China) following the supplier’s instructions.

### ROS measurement

The intracellular accumulation of ROS and Superoxide anion (O_2_^•–^) was monitored using H_2_DCFDA (ThermoFisher Scientific, Waltham, MA, USA) and MitoSOX^TM^ (ThermoFisher Scientific, Waltham, MA, USA), respectively. For ROS detection, IR-L6 myotubes were cultured with 20 μM H_2_DCFDA for 30 min and were then irradiated with 8 J/cm^2^ PBMT. H_2_DCFDA is cleaved by nonspecific esterases and converted to highly fluorescent DCF (Ex/Em, 488/525 nm) upon oxidation by ROS. Fluorescence images were obtained at different point of time using confocal microscopy (LSM510 META; Carl Zeiss Co., Ltd. Jena, Germany). For O_2_^•–^ detection, IR-L6 myotubes were stained with 100 nM MitoTracker Green (MTG; Ex/Em, 488/525 nm; ThermoFisher Scientific, Waltham, MA, USA) for 30 min and 5 μM MitoSOX^TM^ (Ex/Em, 488/580 nm; ThermoFisher Scientific, Waltham, MA, USA) for 10 min. The cells were immediately imaged using confocal microscopy after 8 J/cm^2^ PBMT. All experiments were performed in the dark.

Flow cytometry (FACS) analysis for ROS generation was performed on a FACScanto II flow cytometer (BD Bioscience, San Jose, CA, USA). Cells were incubated with 20 μM H_2_DCFDA (Beyotime Institute Biotechnology, Shanghai, China) for 30 min and then treated with 8 J/cm^2^ PBMT or 100 nM insulin. After 15 min, the cells were harvested and the fluorescence intensity were measured using a FACScanto II flow cytometer. Cells were pre-cultured with 250 μM NAC 1 h before PBMT or insulin treatment. All experiments were performed in the dark.

ROS levels in GM were measured using an Amplex Red Hydrogen Peroxide/Peroxidase Assay Kit (Invitrogen, Carlsbad, CA, USA). In brief, the tissues were lysed in 100 μM Amplex Red reagent and 0.2 U/mL horseradish peroxidase and incubated in the dark for 30 min. Fluorescence was measured using a microplate fluorimeter with excitation at 571 nm and emission at 585 nm.

### Enzymatic activity analysis

Complexes I, II, III, C*c*O and V activities assays was performed as described previously [35]. IR-L6 myotubes after the different doses of PBMT (0, 2, 4, 8, 16 J/cm^2^) were immediately harvested and lysed. Enzymatic activity of C*c*O was detected using a Cytochrome *c* Oxidase Assay Kit (Sigma, St. Louis, MO, USA) according to the manufacturer’s instruction. The enzymatic activity was observed by measuring absorbance of ferrocytochrome *c* at 550 nm using the microplate fluorimeter. Enzymatic activity of complexes I, II, III, and V was performed using MitoCheck Complex I, II, III, and V Activity Assay Kits (Cayman Chemical, MI, USA), respectively, according to the manufacturers’ instructions.

### Single molecule live cell imaging

GFP-GLUT4 and RFP-F-actin plasmids were gifts from Dr. Jeffrey E. Pessin and Dr. Cécile Gauthier Rouvière, respectively. IR-L6 myotubes were transfected with GFP-GLUT4 (Ex/Em, 488/505 nm) and RFP-F-actin (Ex/Em, 543/585 nm) expression plasmids using Lipofectamine 2000 (Invitrogen, Carlsbad, CA, USA) following the manufacturer’s instruction. Twenty-four to 48 hours after transfection, dynamic changes in target protein levels in a single living cell after different treatments were observed immediately by confocal microscopy.

### Immunofluorescence

Immunofluorescence was performed as described previously [46]. In brief, cells were subjected to different treatments, then fixed with 4% paraformaldehyde, followed by permeabilization in methanol, incubated with anti-AKT(S473/4P) (1:300; CST, Danvers, MA, USA) and anti-β-actin (1:300; Santa Cruz Biotechnology, Santa Cruz, CA, USA) at 4° C overnight. Then the cells were incubated for 1.5 h with Goat Anti-Mouse IgG H&L (Alexa Fluor 555) (1:300; Abcam, Cambridge, UK) for β-actin or Goat Anti-Rabbit IgG H&L (Alexa Fluor 488) (1:300; Abcam, Cambridge, UK) for AKT(S473/4P) at room temperature. Nuclei were stained with propidium iodide (PI, Sigma, St. Louis, MO, USA) for 30 min at room temperature before observation. Images were acquired using a confocal microscopy.

### RNA interference

For gene silencing of COXIII, IR-L6 myotubes were transfected with small interfering RNA (siRNA) against COXIII (GenePharma, Shanghai, China) using Lipofectamine 2000. After 36 h, COXIII mRNA in the cells was measured by PCR. Nucleotide sequences of siCOXIII and PCR primers are listed in Supplementary Table 4.

### Immunoblot analysis

For extraction of plasma membrane protein, plasma membrane fractionation was performed as described previously [7]. Cells or tissues were pre-cultured with or without 250 μM NAC or 2 μM API-2 1 h before PBMT. Then they were homogenized, and plasma membrane proteins were obtained using sucrose gradient ultracentrifugation method. For oxidized PTEN analysis, cells and tissues were frozen in liquid nitrogen after different treatment, and lysed with ice-cold RIPA buffer containing 50 mM N-ethylmaleimide (NEM; Aladdin, Shanghai, China), 100 mM PMSF and cocktail set I (protease inhibitor), then centrifuged at 4° C, 12,000 rpm for 20 min [47]. The extracted proteins were separated using immunoblot analysis [25]. Briefly, the extracted proteins were separated in SDS-PAGE, transferred to PVDF membranes (Roche, Penzberg, Germany), and then incubated with primary antibodies and fluorescent secondary antibodies. Detection was performed using a LI-COR Odyssey Scanning Infrared Fluorescence Imaging System (LI-COR, Lincoln, Nebraska, USA). All of the antibodies used are listed in Supplementary Table 4.

### Statistical analysis

Results shown are representative of at least three independent experiments and are presented as mean ± SD. Statistically significant differences between groups were analyzed using one-way ANOVA followed by Student’s *t*-test analysis with SPSS software. A *p*<0.05 was considered statistically significant.

### Data availability statement

Where all the data described in the manuscript are located.

## Supplementary Material

Supplementary Figures

Supplementary Tables 1, 2 and 3

Supplementary Table 4
