# Supplementary material for: Photobiomodulation therapy ameliorates hyperglycemia and insulin resistance by activating cytochrome c oxidase-mediated protein kinase B in muscle
Source: Aging (Albany NY). 2021 Mar 26;13(7):10015–33. doi: 10.18632/aging.202760 (PMC8064177; doi:10.18632/aging.202760)
Supplement: Supplementary Figures [file aging-13-202760-s001.pdf]

SUPPLEMENTARY FIGURES

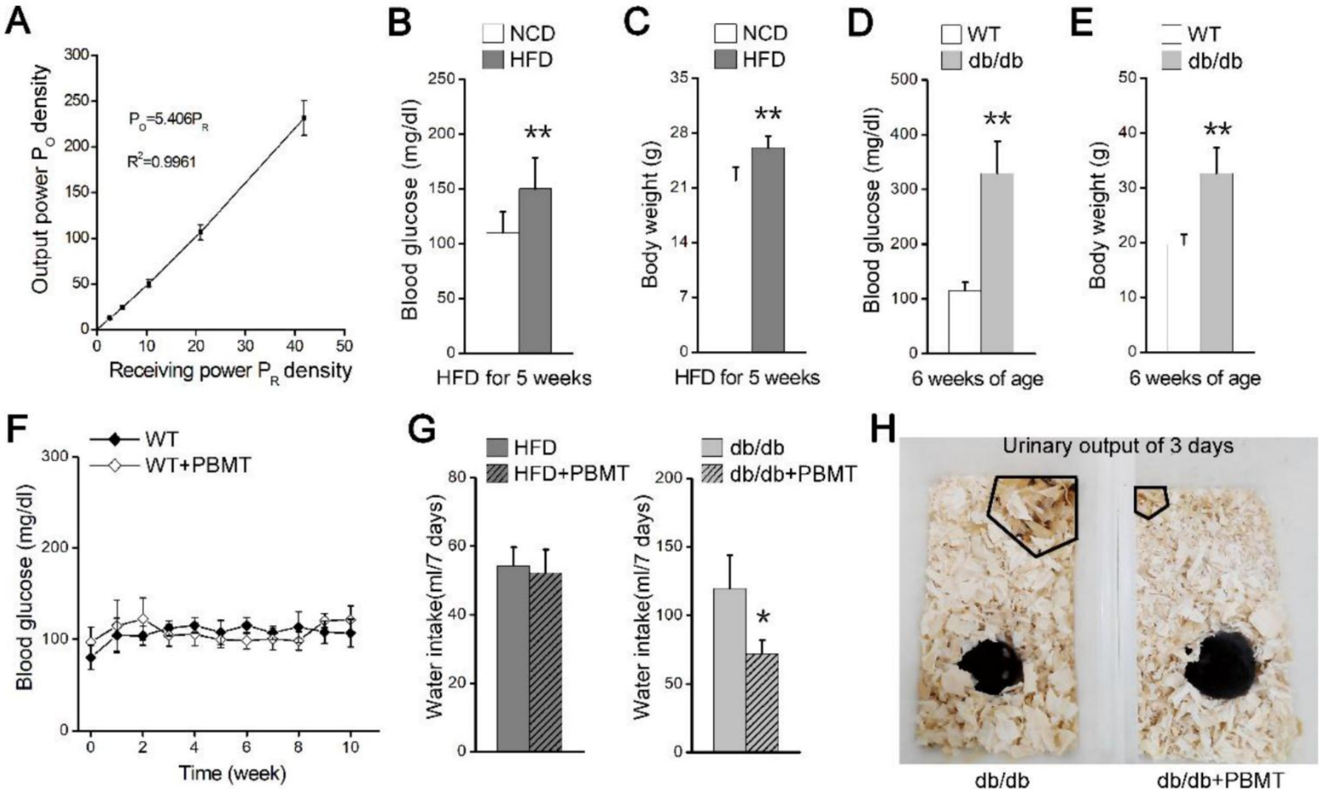

Supplementary Figure 1. PBMT reduces blood glucose and insulin resistance in mouse models of type 2 diabetes. (**A**) The correlation between output power density (Po) and receiving power density (Pr) of Laser Diode (635 nm) passed through the fresh abdominal skin. n = 4. (**B**, **C**) Fasting blood glucose (**B**) and body weight (**C**) in mice fed a NCD (n = 12) and HFD (n = 20) for five weeks. Mean ± SD. ***p* < 0.01 *vs.* the NCD-fed mice (Student's *t*-test). (**D**, **E**) Fasting blood glucose (**D**) and body weight (**E**) in 6-week-old wild-type (WT) and db/db mice after a 12-hour fast. Mean ± SD, n = 6. ***p* < 0.01 *vs.* the WT mice (Student's *t*-test). (**F**) Fasting blood glucose curves of NCD-fed mice with or without PBMT for 10 weeks. Mean ± SD, n = 5. (**G**) Water intake for seven days in HFD-fed and db/db mice treated with or without PBMT for 10 weeks. Mean ± SD, n = 5. **p* < 0.05 *vs.* the PBMT-untreated mice (Student's *t*-test). (**H**) Representative images of urinary output were obtained in db/db mice treated with or without PBMT for 10 weeks.

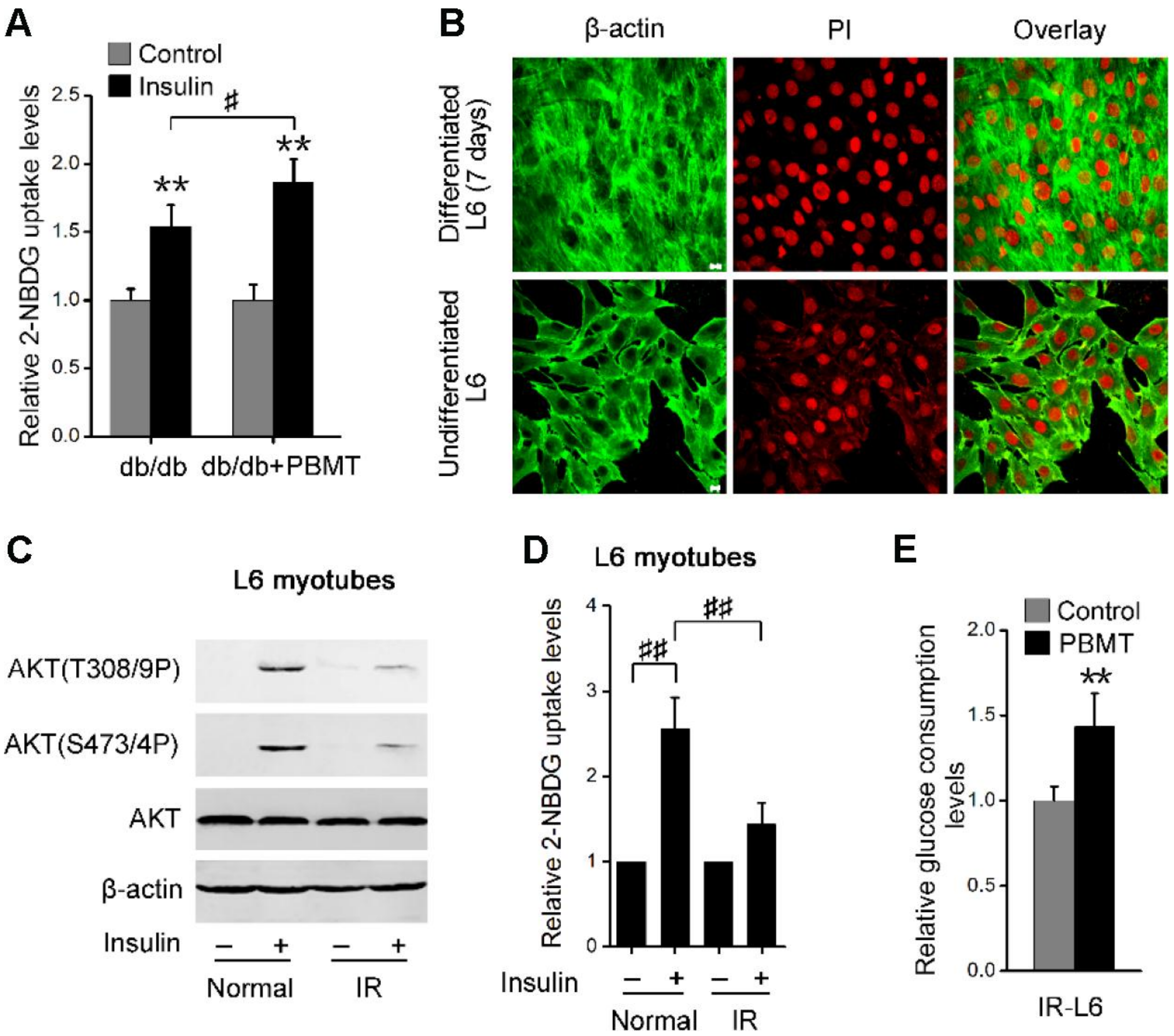

Supplementary Figure 2. PBMT ameliorates metabolic disorders of skeletal muscle in mouse models of type 2 diabetes. (**A**) 2-NBDG uptake in insulin-stimulated GM from db/db mice with or without PBMT for 10 weeks. Mean ± SD, n = 4. $^{**}p < 0.01$ *vs.* the control group; $\#p < 0.05$ *vs.* the indicated group (Student's *t*-test). (**B**) Representative immunofluorescence images of L6 myoblasts differentiation. Cell nuclei (red) were stained with propidium iodide (PI). β-actin (green) was stained with anti-β-actin antibody. Scale bar, 10 μm. (**C**) Immunoblot analysis of AKT phosphorylation in L6 myotubes. IR, insulin resistance. (**D**) 2-NBDG uptake analysis in L6 myotubes. Mean ± SD, n = 4. $\#\#p < 0.01$ *vs.* the indicated group (Student's *t*-test). (**E**) Glucose consumption in conditioned medium from IR-L6 myotubes after PBMT. Mean ± SD, n = 4. $^{**}p < 0.01$ *vs.* the control group (Student's *t*-test).

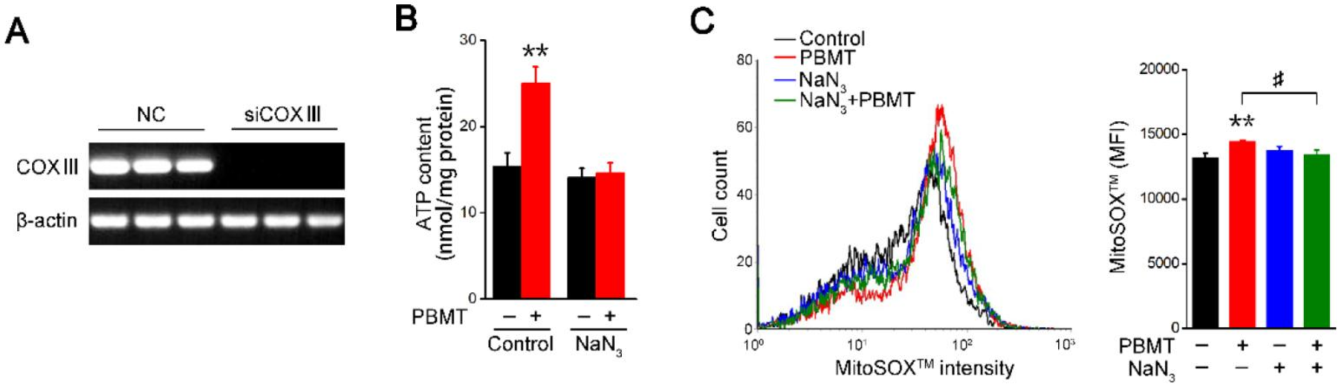

Supplementary Figure 3. PBMT increases ATP and ROS generation by increasing activity of mitochondrial C*c*O. (**A**) PCR analysis of *COXIII* mRNA in IR-L6 myotubes transfected with NC or COXIII siRNA. (**B**) Intracellular ATP content in IR-L6 myotubes 15 min after PBMT. Cells were pre-cultured with NaN_3 (1 mM) 1 h before PBMT. Mean ± SD, n = 4. **$p < 0.01$ *vs.* the PBMT-untreated group (Student's *t*-test). (**C**) FACS analysis of mitochondrial $O_2^{\bullet-}$ generation in IR-L6 myotubes 15 min after PBMT. Cells were pre-cultured with NaN_3 (1 mM) 1 h before PBMT. Mean ± SD, n = 3. **$p < 0.01$ *vs.* the control group; #$p < 0.05$ *vs.* the indicated group (Student's *t*-test).
