# Supplementary material for: Photobiomodulation therapy ameliorates hyperglycemia and insulin resistance by activating cytochrome c oxidase-mediated protein kinase B in muscle
Source: Aging (Albany NY). 2021 Mar 26;13(7):10015–33. doi: 10.18632/aging.202760 (PMC8064177; doi:10.18632/aging.202760)
Supplement: Supplementary Tables 1, 2 and 3 [file aging-13-202760-s002.pdf]

SUPPLEMENTARY TABLES

Supplementary Table 1. Specifications for irradiation parameters.

Parameter (unit)	Value
Center wavelength (nm)	635 nm
Spectral bandwidth (nm)	No (Single wavelength, no bandwidth)
Operating mode	Continuous wave
Frequency (Hz)	No (Continuous wave, no frequency)
Average radiant power (mW)	72.1 mW for mice and 26.7 mW for cells
Polarization	No
Beam divergence	< 2 rad for a Gaussian source
Beam shape	Circular

Supplementary Table 2. Specifications for treatment parameters.

Parameter (unit)	Value
Center wavelength (nm)	635 nm
Spectral bandwidth (nm)	No (Single wavelength, no bandwidth)
Operating mode	Continuous wave
Frequency (Hz)	No (Continuous wave, no frequency)
Average radiant power (mW)	72.1 mW for mice and 26.7 mW for cells
Polarization	No
Beam shape	Circular

Supplementary Table 3. Specifications for treatment parameters.

Parameter (unit)	Value for cells	Value for mice
Beam spot size at target (cm2)	9.2 cm^2	50 cm^2
Exposure duration (sec)	300 s	600 s
Radiant exposure (J/cm2)	8 J/cm^2	43.3 J/cm^2
Radiant energy (J)	73.6 J	2165 J
Number of points irradiated	1	2
Application technique	Without contact	Without contact
Number of treatment sessions	1-2 sessions	70 sessions
Frequency of treatment sessions	If 2 sessions, once every 12 h	Once daily
