# Supplementary material for: Photobiomodulation therapy ameliorates hyperglycemia and insulin resistance by activating cytochrome c oxidase-mediated protein kinase B in muscle
Source: Aging (Albany NY). 2021 Mar 26;13(7):10015–33. doi: 10.18632/aging.202760 (PMC8064177; doi:10.18632/aging.202760)
Supplement: Supplementary Table 4 [file aging-13-202760-s003.docx]

Supplementary Table 4. Key resource table.

| REAGENT or RESOURCE | SOURCE | IDENTIFIER |
| --- | --- | --- |
| Antibodies | | |
| anti-PTEN rabbit monoclonal | CST | #5384 |
| anti-AKT rabbit polyclonal | CST | #9272 |
| anti-AKT (T308/309P) rabbit monoclonal | CST | #13038 |
| anti-AKT (S473/474P) rabbit polyclonal | CST | #9271 |
| anti-GLUT4 rabbit monoclonal | CST | #2213 |
| anti-GSK 3β rabbit monoclonal | CST | #9315 |
| anti-GSK 3β (S9P) rabbit monoclonal | CST | #9322 |
| anti-GS (S641P) rabbit polyclonal | CST | #3891 |
| anti- AMPKα (T172P) rabbit monoclonal | CST | #2535 |
| anti-β-actin mouse monoclonal | Santa Cruz Biotechnology | sc-47778 |
| Goat Anti-Mouse IgG H&L (Alexa Fluor 555) | Abcam | ab150114 |
| Goat Anti-Rabbit IgG H&L (Alexa Fluor 488) | Abcam | ab150077 |
| Goat Anti-Mouse IgG H&L (Alexa Fluor 680) | Abcam | ab175775 |
| Goat Anti-Rabbit IgG H&L (Alexa Fluor 790) | Abcam | ab175781 |
| Chemicals, Peptides, and Recombinant Proteins | | |
| Fetal bovine serum (FBS) | GBICO | 10099-141 |
| Dulbecco’s modified Eagle’s medium (DMEM) | GBICO | 12100046 |
| Horse serum | GBICO | 16050122 |
| Insulin | Tocris Bioscience | 3435 |
| Bovine serum albumin (BSA) | Sigma | V900933 |
| 2-NBDG (2-(N-(7-Nitrobenz-2-oxa-1,3-diazol-4-yl)Amino)-2-Deoxyglucose) | Sigma | 72987 |
| H_2_DCFDA (H2-DCF, DCF) | ThermoFisher Scientific | D399 |
| MitoSOX™ Red Mitochondrial Superoxide Indicator | ThermoFisher Scientific | M36008 |
| MitoTracker Green | ThermoFisher Scientific | M7514 |
| DAF-FM DA | Beyotime Biotechnology | S0019 |
| API-2 | Tocris Bioscience | 2151 |
| Rotenone | Sigma | 45656 |
| Antimycin A | Sigma | A8674 |
| N-acetylcysteine (NAC) | Sigma | A7250 |
| N-Ethylmaleimide (NEM) | Aladdin | E100553 |
| Propidium iodide (PI) | Sigma | 11348639001 |
| Lipofectamine 2000 | Invitrogen | 11668019 |
| Phosphatase inhibitor cocktail tablets | Roche | 04906837001 |
| Triton X-100 | Biosharp | 0694 |
| Tween 20 | Biosharp | 0777 |
| Critical Commercial Assays | | |
| TG assay kit (GPO-POD) | Applygen Technologies Inc. | E1003 |
| Glucose Oxidase Method (GOM) assay kit | Applygen Technologies Inc. | E1011 |
| PIP_3_ ELISA kit | Enzyme-linked Biotechnology Co. | SU-B20592 |
| ATP Assay Kit | Beyotime | S0026 |
| Cytochrome *c* Oxidase Assay Kit | Sigma | CYTOCOX1 |
| MitoCheck Complex I, II, III, and V Activity Assay Kits | Cayman Chemical | 700930/ 700940/  700950/ 701000 |
| Amplex Red Hydrogen Peroxide/Peroxidase Assay Kit | Invitrogen | A22188 |
| Rat Glycogen ELISA kit | Enzyme-linked Biotechnology Co. | 1054086 |
| Mice Glycogen ELISA kit | Enzyme-linked Biotechnology Co. | 1054087 |
| Experimental Models: Cell Lines | | |
| L6 rat myoblasts | ATCC | CRL-1458 |
| A549 | ATCC | CCL-185 |
| Experimental Models: Organisms/Strains | | |
| Mouse (db/db mice): C57BLKS/J-lepr^db^/lepr^db^ | National Resource Center of Model Mice (NRCMM) | N000180 |
| C57BL/6J mice | Beijing Huafukang Bioscience Co. Inc | N/A |
| Oligonucleotides | | |
| *COXⅢ* (Rat) primers: CCTAGTTCCTACCCACGACC/GGAGGCCATGAAATCCCGT | Invitrogen | N/A |
| Actin (Rat) primers: CCGCGAGTACAACCTTCTTG/CAGTTGGTGACAATGCCGTG | Invitrogen | N/A |
| COXIII interference sequences: CCACAAUUCUCCUAUCAUUTT/AAUGAUAGGAGAAUUGUGGTT;  GCAUCAGGAGUCUCAAUUATT/UAAUUGAGACUCCUGAUGCTT;  GGAAUUUACGGCUCAACAUTT/AUGUUGAGCCGUAAAUUCCTT. | GenePharma | N/A |
| Recombinant DNA | | |
| GFP-GLUT4 plasmid | Dr. Jeffrey E. Pessin | N/A |
| RFP-F-actin plasmid | Dr. Georges Bismuth | N/A |
| Software and Algorithms | | |
| Zeiss LSM Image Browser Version 4,2,0,121 | Carl Zeiss Jena, Germany | http://www.filedudes.com/Zeiss_LSM_Image_Browser-download-172844.html |
| Image J 1.49v | NIH, USA | http://imagej.nih.gov/ij |
| FCS Express Version 3 | De Novo Software, Canada | http://www.denovosoftware.com/ |
| Image Studio Lite Version 5.2.5 | Li-Cor, USA | https://www.licor.com/bio/products/software/image_studio_lite/ |
| Microcal Origin 6.0 | Microcal Software, Inc., USA | http://www.microcal.com |
| SPSS Statistics 20.0 | IBM, USA | http://www-01.ibm.com/support/docview.wss?uid=swg24029274 |
| Other | | |
| PVDF membranes | Roche | 15389600 |
| TGX™ FastCast™ Acrylamide Starter Kit | Bio-rad | 1610174 |
| Prestained Protein Ladder | ThermoFisher Scientific | 26616 |
| High fatty diet (60%) | Beijing Huafukang Bioscience Co. Inc. | D12492 |
